# Genotypic and Allelic Variability in *CYP19A1* among Populations of African and European Ancestry

**DOI:** 10.1371/journal.pone.0117347

**Published:** 2015-02-03

**Authors:** Athena Starlard-Davenport, Mohammed S. Orloff, Ishwori Dhakal, Rosalind B. Penney, Susan A. Kadlubar

**Affiliations:** 1 Department of Medical Genetics, University of Arkansas for Medical Sciences, Little Rock, AR 72205, United States of America; 2 Department of Epidemiology, College of Public Health, University of Arkansas for Medical Sciences, Little Rock, AR 72205, United States of America; 3 Department of Biostatistics, University of Arkansas for Medical Sciences, Little Rock, AR 72205, United States of America; 4 Department of Environmental and Occupational Health, College of Public Health, University of Arkansas for Medical Sciences, Little Rock, AR 72205, United States of America; Universitat Pompeu Fabra, SPAIN

## Abstract

*CYP19A1* facilitates the bioconversion of estrogens from androgens. *CYP19A1* intron single nucleotide polymorphisms (SNPs) may alter mRNA splicing, resulting in altered CYP19A1 activity, and potentially influencing disease susceptibility. Genetic studies of *CYP19A1* SNPs have been well documented in populations of European ancestry; however, studies in populations of African ancestry are limited. In the present study, ten ‘candidate’ intronic SNPs in *CYP19A1* from 125 African Americans (AA) and 277 European Americans (EA) were genotyped and their frequencies compared. Allele frequencies were also compared with HapMap and ASW 1000 Genomes populations. We observed significant differences in the minor allele frequencies between AA and EA in six of the ten SNPs including rs10459592 (p<0.0001), rs12908960 (p<0.0001), rs1902584 (p = 0.016), rs2470144 (p<0.0001), rs1961177 (p<0.0001), and rs6493497 (p = 0.003). While there were no significant differences in allele frequencies between EA and CEU in the HapMap population, a 1.2- to 19-fold difference in allele frequency for rs10459592 (p = 0.004), rs12908960 (p = 0.0006), rs1902584 (p<0.0001), rs2470144 (p = 0.0006), rs1961177 (p<0.0001), and rs6493497 (p = 0.0092) was observed between AA and the Yoruba (YRI) population. Linkage disequilibrium (LD) blocks and haplotype clusters that is unique to the EA population but not AA was also observed. In summary, we demonstrate that differences in the allele frequencies of *CYP19A1* intron SNPs are not consistent between populations of African and European ancestry. Thus, investigations into whether *CYP19A1* intron SNPs contribute to variations in cancer incidence, outcomes and pharmacological response seen in populations of different ancestry may prove beneficial.

## Introduction

Cytochrome P450 19A1 (*CYP19A1*) encodes the enzyme aromatase, which catalyzes the conversion of the C19 androgens, androstenedione and testosterone, to estrone and estradiol, respectively [1,2]. Specific single nucleotide polymorphisms (SNPs) in the intronic regions of *CYP19A1* have been shown to play a role in altering regulation of transcription and/or splicing of *CYP19A1*, producing different enzyme products with variable enzymatic activity compared to the normal gene product [3,4]. Studies have identified SNPs in *CYP19A1* that are associated with cancer risk primarily in European Americans (EA), North Indian and Chinese populations [5,6]. Variations in the allele frequencies of several *CYP19A1* SNPs and their haplotype distributions, especially rs10459592, rs749292, and rs6493497, have also been documented within South Indian, Korean, Hawaiian, Japanese, Latina and populations of European descent within the United States [7–9]. It is thus likely that ancestral differences in the frequencies of functional *CYP19A1* SNPs can influence disease susceptibility and risk prediction. However, genetic studies of *CYP19A1* SNPs in populations of African ancestry are limited.

Human *CYP19A1* (Genbank accession number: NC_000015.10) is mapped to the positive strand of the long arm of chromosome 15 at 15q21.2 at chromosomal location 15: chr15:51,222,349–51,338,598. *CYP19A1* is approximately 116 kb long and comprises nine protein coding exons and a number of alternative non-coding first exons that regulate tissue-specific expression [10]. *S*everal genetic variants of *CYP19A1* are localized within the introns. Intronic SNPs can potentially influence mRNA splicing, leading to *CYP19A1* dysfunction. Variability in the frequencies of functional *CYP19A1* SNPs can impact a multiplicity of functional elements, including intron splice enhancers and silencers that regulate alternative splicing, trans-splicing elements [11], and other regulatory elements. Several intronic SNPs located within the regulatory regions of *CYP19A1* have been shown to influence estrogen-dependent disease risk, serum estrogen levels and/or aromatase production [12–16]. Furthermore, SNPs located within introns of *CYP19A1* have been implicated in the development of multicentric adenocarcinomas in the peripheral lung [17], Alzheimer’s disease [18], and neuroprotection through the neuroprotective actions of estrogens [19].

In light of these considerations, we hypothesized that the frequency distribution of *CYP19A1* intron SNPs that are associated with disease risk would differ between populations of European and African ancestry. To test our hypothesis, we determined the allele frequencies of ten candidate *CYP19A1* SNPs, constructed haplotypes, and assessed their distributions in populations of European and African ancestry from Arkansas.

## Materials and Methods

### Study Subjects

The study population consisted of 407 unrelated, healthy AA (n = 125) and EA (n = 277) volunteers who were recruited from 1998 to 2003 at the University of Arkansas for Medical Sciences University Hospital in Little Rock, Arkansas. Of the 125 AA subjects, 55 were male and 70 were female, and out of 277 EA subjects 142 were male and 135 were female. Age range among AA subjects was 22–75 years of age. The mean age for AA males was 58.7 ± 15.6 and for AA females was 52.3 ± 14.0. Among EA subjects, age of subjects ranged from 21–85 years of age. The mean age for EA males was 61.6 ± 14.6 and for EA females was 56.4 ± 15.3. There was no significant difference in age between AA and EA by sex (p = 0.15) or by ethnicity (p = 0.38). The study was performed according to the Declaration of Helsinki and approved by the University of Arkansas for Medical Sciences Institutional Review Board. Subjects provided their written informed consent prior to study participation.

### *CYP19A1* SNP Selection and Genotyping

Ten *CYP19A1* SNPs genotyped in this study were selected based upon their previously published associations with cancer risk and outcomes (Table 1), predicted localization within regulatory binding regions, and/or their predicted association with regulatory proteins involved in pre-mRNA processing, mRNA metabolism and transport, and gene expression (Table 2). Identifying possible function roles of variants in HaploReg version 2 [20], Human Splicing Finder (version 2.4.1) [21], and TFSEARCH [22] was performed using the dbSNP rs number or 75bp nucleotide sequences upstream and downstream of the *CYP19A1* SNP to identify potential target sites containing the test SNP, proteins that regulate expression of *CYP19A1*, and transcription factor binding sites that had a probability score ≥90%.

**Table 1:** Clinical implications of *CYP19A1* SNPs analyzed.

*CYP19A1* SNP	Clinical Implication	Reference
rs10459592	Predictor of clinical outcomes and adverse events associated with letrozole use in patients with metastatic breast cancer	[25]
rs12591359	Significantly associated with increased risk of colon cancer development	[26]
rs749292	Associated with an increase in circulating estrogen levels in postmenopausal women and increased endometrial cancer risk	[13], [24]
rs2470152	Associated with hormone estradiol levels; Significantly associated with increased risk of vertebral fractures; Associated with worse Glasgow Outcome Scale-6 scores after traumatic brain injury	[29], [30]
rs1902584	Associated with increased colorectal cancer risk in women; Associated with hormone estradiol levels in overweight postmenopausal women	[6, 31]
rs2470144	Associated with worse Glasgow Outcome Scale-6 scores after traumatic brain injury; Significantly associated with increased annual sagittal maxillary growth and mandibular growth in boys	[30, 32]
rs1961177	Significantly associated with an increased likelihood of a MSI+ colon tumor; Significantly associated with increased aortic diameter in men	[26, 33]
rs6493497	Significantly associated with a greater change in aromatase activity after AI treatment and higher plasma estradiol levels in patients pre-AI and post-AI treatment	[34]

**Table 2:** Characteristics of *CYP19A1* SNPs and predicted binding sites and associated regulatory proteins.

*CYP19A1* SNP	Location	Position	Nucleotide change	SNP Genotyping Assay ID	Transcription Factor Binding Sites	Associated Proteins
rs10459592	Intron	51536141	T>G	C_30576547	AP-1; CAP; GCN4	SC35; hnRNP A1; NHEK; HMEC
rs12591359	Intron	51539368	G>A	C_32071405	HSF; CAP; ADR1	SF2/ASF (IgM-BRCA1); ATF3; Maf; SRp55
rs12908960	Intron	51545860	G>A	C_32071412	HSF; STRE; ADR1	hnRNP A1; HMEC; CFOS
rs11856927	Intron	51548705	G>T	C_11301470	ADR1; CF1; HSF; CAP	SC35
rs749292	Intron	51558731	G>A	C_8801261	HSF; CdxA; ADR1; HSF2	SF2/ASF; 9G8; HSMM
rs2470152	Intron	51594972	C>T	C_3060064	HSF	SF2/ASF; 9G8; CTCF; NFKB
rs1902584	Intron	51611654	A>T	C_1664181	HSF; SOX-5; OCT-1	9G8; Tra2-β
rs2470144	Intron	51621725	A>G	C_3060076	ADR1; CAP	SRp55
rs1961177	Intron	51625078	C>T	C_27173536	HSF; CAP; NKX-2; SRY	Tra2-β; Huvec; STAT3
rs6493497	5’ UTR	51630835	G>A	C_29374681	CAP; TATA; CDXA	-

Genotyping of *CYP19A1* SNPs was conducted using the TaqMan allelic discrimination assay on ABI PRISM 7900 HT platform (Life Technologies, CarIsbad, USA) according to the manufacturer’s recommendations and established quality control measures for reliable genotyping results in the laboratory. In each 384-well reaction plate, two negative controls and positive DNA controls with known SNP genotype at the *CYP19A1* were added for quality control. In addition, each DNA sample (20ng) was genotyped in duplicate. The genotype data was analyzed by using SDS 2.3 Allelic Discrimination Software (Applied Biosystems).

### Statistical Analyses

Genotype and allele frequencies with the 95% confidence interval (95% CI) for each SNP were calculated using SAS version 9.2 (SAS Institute, Cary, NC) and PLINK v1.07 (http://pngu.mgh.harvard.edu/purcell/plink/) [23]. Ethnic differences in genotype frequency for each SNP were compared using Pearson Chi-Square test. A test for the deviation from the Hardy-Weinberg equilibrium was performed for all the SNPs included in the study. Any additional analyses were done using SAS version 9.2 (SAS Institute, Cary, NC). Linkage disequilibrium (Pairwise linkage disequilibrium (LD) (D’) for the SNPs was evaluated and visualized in HAPLOVIEW software (Version 4.2) [24]. LD), between pairs of alleles at different loci (i.e. SNPs), was calculated through the computing of the standardized LD value (D’). D’ is the normalization of the LD, dividing it by the theoretical maximum value for the observed allele frequencies (D’ = LD/LDmax). |D’| = 1 indicates complete LD and D’ = 0 corresponds to total absence of LD. Pairwise LD for the SNPs was visualized in HAPLOVIEW software (Version 4.2) [24]. After assessing the LD patterns, non-missing genotype frequencies from all SNPs were used to reconstruct haplotypes and estimate their respective frequencies in the two populations using the PHASE v2.1.1 program [25]. This program has been shown to be ideal to generate haplotypes from multiple loci or long DNA sequence stretches. We ran PHASE in three different settings i) 100 iterations (default setting) ii) 500 iterations [i.e. with option –X5] iii) 1000 iterations [i.e. with option –X10]. Also the pseudo-random number generator for second and third run were changed with –S option (i.e. for second run: –S211 and for the third run:–S3253). We observed estimated haplotype frequencies across different runs were fairly consistent.

## Results

### Comparison of *CYP19A1* genotype and allele frequencies between AA and EA subjects from Arkansas

We genotyped ten candidate *CYP19A1* SNPs in AA and EA populations. Significant differences in the minor allele frequencies (MAF) between EA and AA populations in six of ten *CYP19A1* SNPs were observed (Table 3). Specifically, a significant difference in the MAF between AA and EA was observed for rs10459592 (p<0.0001), rs12908960 (p<0.0001), rs1902584 (p = 0.016), rs2470144 (p<0.0001), rs1961177 (p<0.0001), and rs6493497 (p = 0.003). There was no difference in the MAF between AA and EA for the remaining four SNPS, i.e. rs12591359 (p = 0.891), rs11856927 (p = 0.439), rs749292 (p = 0.434), or rs2470152 (p = 0.19). All SNPs, except for rs1902584, were consistent with HWE in both populations. Genotypes generated from rs1902584 were confirmed to be free from genotyping error.

**Table 3:** Ethnic differences in *CYP19A1* minor allele frequency distribution across populations of European (EA) and African (AA) ancestry.

	Minor Allele Frequency (%)		
CYP19A1 SNP	EA	AA	P value
rs10459592	56.5	38.4	<.0001***
rs12591359	39.2	39.3	.891
rs12908960	45.6	15.1	<.0001***
rs11856927	42.9	44.6	.439
rs749292	47.3	48.0	.434
rs2470152	46.2	46.3	0.19
rs1902584	11.2	15.5	0.016*
rs2470144	46.8	16.8	<.0001***
rs1961177	15.4	38.4	<.0001***
rs6493497	15.2	23.9	0.003**

*p<0.05;

**p<0.01;

***p<0.001

We also compared the *CYP19A1* MAF between EA and AA populations with those from HapMap and the ASW 1000 Genomes population. Population allele and genotype frequencies for all the *CYP19A1* SNPs analyzed in this study are summarized in Table 4. The HapMap and other populations analyzed included Utah residents with Northern and Western European ancestry (CEU), Yoruba in Ibadan, Nigeria (YRI), Han Chinese in Beijing, China (HCB), Japanese in Tokyo, Japan (JPT), and Gujarat Indians in Houston, Texas (GIH) that were previously reported according to the NCBI Entrez database, as well as South Indians (SI) and Koreans (KOR). Significant differences in the MAF for rs12591359, rs12908960, rs749292, and rs2470144 were observed between AA and EA populations and the SI, HCB, JPT, YRI, and GIH populations. When comparing EA in our study with CEU, no significant differences in the MAF between the two populations was evident (p = 0.871). However, a 1.2-fold to 19-fold difference in the MAF for rs10459592 (p = 0.004), rs1902584 (p<0.0001), rs2470144 (p = 0.0006), and rs12908960 (p = 0.0006) was observed between AA and YRI, but not between AA and ASW populations, which are each populations of African ancestry. There were no significant differences in the allele and genotype frequencies for the *CYP19A1* SNPs analyzed in this study by sex (data not shown).

**Table 4:** Allele and genotype frequencies of the *CYP19A1* SNPs in various populations.

SNP	Pop. (N)	Genotype frequency (95% CI)			Allele frequency (95% CI)		HWE ρ value
rs10459592		TT	TG	GG	T	**G**	
	AA(121)^a^	39.7 (31.0–48.4)	43.8 (34.9–52.6)	16.5 (9.89–23.1)	61.6 (52.9–70.3)	38.4 (29.8–47.1)	0.48
	EA(263)^a^	19.4 (14.6–24.2)	48.3 (42.3–54.3)	32.3 (26.6–38.0)	43.5 (37.5–49.5)	56.5 (50.5–62.5)	0.89
	CEU(226)^b^	19.5 (14.3–24.6)	39.8 (33.4–46.2)	40.7 (34.3–47.1)	39.4 (34.9–43.9)	60.6 (56.1–65.1)	0.10
	YRI(226)^b^	55.8 (49.3–62.2)	34.5 (28.3–40.7)	9.70 (6.2–14.4)	73.0 (68.9–77.1)	27.0 (22.9–31.1)	0.28
	HCB(86)^b^	27.9 (18.8–38.6)	46.5 (35.7–57.6)	25.6 (16.8–36.1)	51.2 (43.7–58.6)	48.8 (41.4–56.3)	0.65
	JPT(172)^b^	39.5 (32.2–46.8)	44.2 (36.8–51.6)	16.3 (10.8–21.8)	61.6 (56.5–66.8)	38.4 (33.2–43.5)	0.58
	GIH(176)^b^	21.6 (15.5–27.7)	53.4 (46.0–60.8)	25.0 (18.6–31.4)	48.3 (43.1–53.5)	51.7 (46.5–56.9)	0.52
	SI(163)^c^	43.6 (35.9–51.2)	45.4 (37.8–53.0)	11.0 (6.2–15.9)	66.3 (61.1–71.4)	33.7 (28.6–38.9)	0.84
	KOR(50)^d^	24.0 (13.1–38.2)	54.0 (39.3–68.2)	22.0 (11.5–36.0)	51.0 (40.8–61.1)	49.0 (38.9–59.2)	0.56
	ASW(122)^e^	37.7 (37.2–38.2)	57.4 (56.8–58.0)	4.90 (4.82–4.98)	66.4 (65.8–67.0)	33.6 (33.2–34.0)	<0.05
rs12591359		GG	GA	AA	G	**A**	
	AA (103)^a^	36.9 (27.6–46.2)	47.6 (38.0–57.2)	15.5 (8.51–22.5)	60.7 (51.3–70.1)	39.3 (29.9–48.7)	0.96
	EA (106)^a^	38.7 (29.4–48.0)	44.3 (34.8–53.8)	17.0 (9.85–24.2)	60.8 (51.5–70.1)	39.2 (29.9–48.5)	0.54
	CEU(224)^b^	42.0 (35.5–48.5)	40.2 (33.8–46.6)	17.9 (12.9–22.9)	62.1 (55.7–68.5)	37.9 (31.5–44.3)	0.15
	YRI (226)^b^	38.9 (32.5–45.3)	47.8 (41.3–54.3)	13.3 (8.87–17.7)	62.8 (56.5–69.1)	37.2 (30.9–43.5)	0.80
	HCB (86)^b^	20.9 (12.3–29.5)	37.2 (27.0–47.4)	41.9 (31.5–52.3)	39.5 (29.2–49.8)	60.5 (50.2–70.8)	0.15
	JPT (172)^b^	9.30 (4.96–13.6)	45.3 (37.9–52.7)	45.3 (37.9–52.7)	32.0 (25.0–39.0)	68.0 (61.0–75.0)	0.75
	GIH(176)^b^	37.5 (30.3–44.7)	43.2 (35.9–50.5)	19.3 (13.5–25.1)	59.1 (51.8–66.4)	40.9 (33.6–48.2)	0.32
	ASW(122)^e^	39.3 (38.8–39.8)	42.6 (42.1–43.1)	18.0 (17.7–18.3)	60.7 (60.1–61.3)	39.3 (38.8–39.8)	0.27
rs12908960		GG	GA	AA	**G**	**A**	
	AA (83)^a^	75.9 (66.7–85.1)	18.1 (9.82–26.4)	6.02 (0.90–11.1)	84.9 (77.2–92.6)	15.1 (7.40–22.8)	0.18
	EA (225)^a^	31.6 (25.5–37.7)	45.8 (39.3–52.3)	22.7 (17.2–28.2)	54.4 (47.9–60.9)	45.6 (39.1–52.1)	0.28
	CEU(226)^b^	26.5 (20.7–32.3)	45.1 (38.6–51.6)	28.3 (22.4–34.2)	49.1 (42.6–55.6)	50.9 (44.4–57.4)	0.32
	YRI (226)^b^	92.0 (88.5–95.5)	7.10 (3.75–10.4)	0.90 (0.33–2.13)	95.6 (92.9–98.3)	4.40 (1.73–7.07)	0.10
	HCB (86)^b^	20.9 (12.3–29.5)	48.8 (38.2–59.4)	30.2 (20.5–39.9)	45.3 (34.8–55.8)	54.7 (44.2–65.2)	0.92
	JPT (172)^b^	43.0 (35.6–50.4)	40.7 (33.4–48.0)	16.3 (10.8–21.8)	63.4 (56.2–70.6)	36.6 (29.4–43.8)	0.25
	GIH(176)^b^	47.7 (40.3–55.1)	37.5 (30.3–44.7)	14.8 (9.55–20.0)	66.5 (59.5–73.5)	33.5 (26.5–40.5)	0.15
	ASW(122)^e^	77.0 (76.4–77.6)	21.3 (21.0–21.6)	1.60 (1.57–1.62)	87.7 (87.2–88.2)	12.3 (12.1–12.5)	0.74
rs11856927		GG	GT	TT	T	**G**	
	AA (121)^a^	16.5 (9.89–23.1)	56.2 (47.4–65.0)	27.3 (19.4–35.2)	55.4 (46.5–64.3)	44.6 (35.7–53.5)	0.15
	EA (262)^a^	18.3 (13.6–23.0)	49.2 (43.1–55.3)	32.4 (26.7–38.1)	57.1 (51.1–63.1)	42.9 (36.9–48.9)	0.92
	CEU(116)^b^	19.0 (11.9–26.1)	48.3 (39.2–57.4)	32.8 (24.3–41.3)	56.9 (47.9–65.9)	43.1 (34.1–52.1)	0.89
	YRI (120)^b^	25.0 (17.3–32.7)	38.3 (29.6–47.0)	36.7 (28.1–45.3)	55.8 (46.9–64.7)	44.2 (35.3–53.1)	0.10
	HCB (90)^b^	33.3 (23.6–43.0)	51.1 (40.8–61.4)	15.6 (8.10–23.1)	41.1 (30.9–51.3)	58.9 (48.7–69.1)	0.51
	JPT (90)^b^	15.6 (8.10–23.1)	35.6 (25.7–45.5)	48.9 (38.6–59.2)	66.7 (57.0–76.4)	33.3 (23.6–43.0)	0.20
	ASW(122)^e^	21.3 (21.0–21.6)	47.5 (46.9–48.1)	31.1 (30.7–31.5)	54.9 (54.3–55.5)	45.1 (44.6–45.6)	0.69
rs749292		GG	GA	AA	G	**A**	
	AA (101)^a^	23.8 (14.5–30.9)	56.4 (46.7–66.1)	19.8 (12.0–27.6)	52.0 (42.3–61.7)	48.0 (38.3–57.7)	0.15
	EA (75)^a^	22.7 (38.0–60.6)	49.3 (38.0–60.6)	28.0 (17.8–38.2)	47.3 (36.0–58.6)	52.7 (41.4–64.0)	0.95
	CEU(226)^b^	31.9(25.8–37.9)	45.1(38.6–51.6)	23.0(17.5–28.5)	54.4(49.8–59.0)	45.6(41.0–50.2)	0.40
	YRI (226)^b^	25.5(19.2–30.4)	48.7(42.2–55.2)	27.0(20.8–32.3)	49.1(44.5–53.7)	50.9(46.3–55.5)	0.69
	HCB (86)^b^	9.30(4.10–17.5)	53.5(42.4–64.3)	37.2(27.0–48.3)	36.0(28.9–43.2)	64.0(56.8–71.1)	0.10
	JPT (172)^b^	38.3(31.1–45.6)	47.7(40.2–55.1)	14.0(8.80–19.1)	62.2 (57.1–67.3)	37.8(32.7–42.9)	0.93
	GIH(176)^b^	39.8(32.5–47.0)	43.2(35.9–50.5)	17.0(11.5–22.6)	61.4(56.3–66.5)	38.6(33.5–43.7)	0.40
	SI (163)c	57.7(50.1–65.3)	35.0(27.6–42.3)	7.30(3.8–12.5)	75.1(70.5–79.8)	24.9(20.2–29.5)	0.61
	ASW(122)^e^	29.5 (29.1–29.9)	55.7 (55.1–56.3)	14.8 (14.6–15.0)	57.4 (56.8–58.0)	42.6 (42.1–43.1)	0.14
rs2470152		CC	CT	TT	T	**C**	
	AA (122)^a^	28.7 (20.7–36.7)	50.8 (41.9–59.7)	20.5 (13.3–27.7)	54.1 (45.3–62.9)	46.3 (37.1–54.7)	0.86
	EA (265)^a^	19.6 (14.8–24.4)	53.2 (47.2–59.2)	27.2 (21.8–32.6)	46.2 (40.2–52.2)	53.8 (47.8–59.8)	0.27
	CEU(226)^b^	31.0 (25.0–37.0)	47.8 (41.3–54.3)	21.2 (15.9–26.5)	45.1 (38.6–51.6)	54.9 (48.4–61.4)	0.75
	YRI (226)^b^	16.8 (11.9–21.7)	46.0 (39.5–52.5)	37.2 (30.9–43.5)	60.2 (53.8–66.6)	39.8 (33.4–46.2)	0.79
	HCB (86)^b^	32.6 (22.7–42.5)	51.2 (40.6–61.8)	16.3 (8.49–24.1)	41.9 (31.5–52.3)	58.1 (47.7–68.5)	0.56
	JPT (172)^b^	29.1 (22.3–35.9)	41.9 (34.5–49.3)	29.1 (22.3–35.9)	50.0 (42.5–57.5)	50.0 (42.5–57.5)	0.15
	ASW(122)^e^	21.3 (21.0–21.6)	49.2 (48.6–49.8)	29.5 (29.1–29.9)	54.1 (53.5–54.7)	45.9 (45.4–46.4)	0.97
rs1902584		AA	AT	TT	A	**T**	
	AA (100)^a^	82.0 (74.5–89.5)	6.00 (1.35–10.7)	12.0 (5.63–18.4)	85.0 (78.0–92.0)	15.0 (8.00–22.0)	<0.05
	EA (272)^a^	83.1 (78.6–87.6)	12.1 (8.22–16.0)	4.78 (2.24–7.32)	89.1 (85.4–92.8)	10.8 (7.11–14.5)	<0.05
	CEU(224)^b^	83.0 (78.1–87.9)	16.1 (11.3–20.9)	0.90 (0.34–2.14)	91.1 (87.4–94.8)	8.90 (5.17–12.6)	0.38
	YRI (226)^b^	89.4 (85.4–93.4)	10.6 (6.59–14.6)	0	94.7 (91.8–97.6)	5.30 (2.38–8.22)	0.60
	HCB (86)^b^	72.1 (62.6–81.6)	25.6 (16.4–34.8)	2.30 (0.87–5.47)	84.9 (77.3–92.5)	15.1 (7.53–22.7)	0.92
	JPT (170)^b^	72.9 (66.2–79.6)	24.7 (18.2–31.2)	2.40 (0.10–4.70)	85.3 (80.0–90.6)	14.7 (9.38–20.0)	1.0
	GIH(176)^b^	71.6 (64.9–78.3)	22.7 (16.5–28.9)	5.70 (2.27–9.13)	83.0 (77.5–88.5)	17.0 (11.5–22.5)	0.10
	ASW(122)^e^	85.2 (84.7–85.7)	14.8 (14.6–15.0)	0	92.6 (92.2–93.0)	7.40 (7.29–7.51)	0.45
rs2470144		GG	GA	AA	G	**A**	
	AA (113)^a^	71.7 (63.4–80.0)	23.0 (15.2–30.8)	5.30 (1.17–9.43)	83.2 (76.3–90.1)	16.8 (9.91–23.7)	0.08
	EA (254)^a^	25.2 (19.9–30.5)	43.3 (37.2–49.4)	31.5 (25.8–37.2)	53.1 (47.0–59.2)	46.9 (40.8–53.0)	0.20
	CEU(226)^b^	31.0 (25.0–37.0)	47.8 (41.3–54.3)	21.2 (15.9–26.5)	54.9 (48.4–61.4)	45.1 (38.6–51.6)	0.69
	YRI (226)^b^	81.4 (76.3–86.5)	18.6 (13.5–23.7)	0	90.7 (86.9–94.5)	9.30 (5.51–13.1)	0.66
	HCB (86)^b^	30.2 (20.5–39.9)	46.5 (36.0–57.0)	23.3 (14.4–32.2)	53.5 (43.0–64.0)	46.5 (36.0–57.0)	0.75
	JPT (172)^b^	31.4 (24.5–38.3)	54.7 (47.3–62.1)	14.0 (8.81–19.2)	58.7 (51.3–66.1)	41.3 (33.9–48.7)	0.22
	GIH(176)^b^	14.8 (9.55–20.0)	48.9 (41.5–56.3)	36.4 (29.3–43.5)	39.2 (32.0–46.4)	60.8 (53.6–68.0)	0.75
	ASW(122)^e^	63.9 (63.3–64.5)	34.4 (34.0–34.8)	1.60 (1.57–1.63)	81.1 (80.5–81.7)	18.9 (18.6–19.2)	0.12
rs1961177		CC	CT	TT	C	**T**	
	AA (124)^a^	39.5 (30.9–48.1)	45.2 (36.4–54.0)	15.3 (8.96–21.6)	62.1 (53.6–70.6)	38.4 (29.4–46.4)	0.70
	EA (272)^a^	71.7 (66.3–77.1)	26.1 (20.9–31.3)	2.20 (0.46–3.94)	84.7 (80.4–89.0)	15.3 (11.0–19.6)	0.83
	CEU(226)^b^	71.7 (65.8–77.6)	26.5 (20.7–32.3)	1.80 (0.07–3.53)	85.0 (80.3–89.7)	15.0 (10.3–19.7)	0.75
	YRI (226)^b^	29.2 (23.3–35.1)	47.8 (41.3–54.3)	23.0 (17.5–28.5)	53.1 (46.6–59.6)	46.9 (40.4–53.4)	0.75
	HCB (86)^b^	62.8 (52.6–73.0)	32.6 (22.7–42.5)	4.70 (0.23–9.17)	79.1 (70.5–87.7)	20.9 (12.3–29.5)	0.96
	JPT (172)^b^	62.2 (55.0–69.4)	33.7 (26.6–40.8)	3.50 (0.75–6.25)	79.7 (73.7–85.7)	20.3 (14.3–26.3)	0.75
	GIH(176)^b^	68.2 (61.3–75.1)	25.0 (18.6–31.4)	6.80 (3.08–10.5)	80.7 (74.9–86.5)	19.3 (13.5–25.1)	0.10
	ASW(122)^e^	37.7 (37.2–38.2)	49.2 (48.6–49.8)	13.1 (12.9–13.3)	62.3 (61.7–62.9)	37.7 (37.2–38.2)	0.61
rs6493497		GG	GA	AA	G	**A**	
	AA (96)^a^	56.3 (46.4–66.2)	40.6 (30.8–50.4)	3.13 (0.35–6.61)	76.6 (68.1–85.1)	23.4 (14.9–31.9)	0.19
	EA (270)^a^	74.1 (68.9–79.3)	22.6 (17.6–27.6)	3.33 (1.19–5.47)	85.4 (81.2–89.6)	14.6 (10.4–18.8)	0.40
	CEU(224)^b^	72.3 (66.5–78.2)	25.9 (20.2–31.6)	1.80 (1.78–1.82)	85.3 (82.0–88.5)	14.7(11.5–18.0)	0.46
	YRI (226)^b^	52.2 (45.7–58.7)	38.9 (32.6–45.3)	8.80 (5.50–13.3)	71.7 (67.5–75.8)	28.3 (24.2–32.5)	0.78
	HCB (86)^b^	62.8 (51.7–73.0)	32.6 (22.8–43.5)	4.70 (1.30–7.50)	79.1 (73.0–85.1)	20.9 (14.9–27.0)	0.96
	JPT (170)^b^	63.5 (56.3–70.8)	32.9 (25.9–40.0)	3.50 (1.30–7.50)	80.0 (75.7–84.3)	20.0 (15.7–24.3)	0.66
	GIH(176)^b^	68.2 (61.3–75.1)	25.0 (18.6–31.4)	6.80 (3.60–11.6)	80.7 (76.6–84.8)	19.3 (15.2–23.4)	0.10
	SI (163)^c^	39.9 (32.4–47.4)	48.5 (40.8–56.1)	11.6 (6.70–16.6)	64.1 (58.9–69.3)	35.9 (30.7–41.1)	0.51
	KOR (50)^d^	48.0 (33.7–62.6)	42.0 (28.2–56.8)	10.0 (3.30–21.8)	69.0 (59.0–77.9)	31.0 (22.1–41.0)	0.89
	ASW(122)^e^	60.7 (60.0–61.3)	34.4 (34.0–34.8)	4.90 (4.82–4.98)	77.9 (77.3–78.5)	22.1 (21.8–22.4)	0.76

African Americans; EA: European Americans; CEU: Utah residents with Northern and European ancestry; YRI: Yoruba in Ibadan, Nigeria; HCB: Hans Chinese in Beijing, China; JPT: Japanese in Tokyo, Japan; and GIH Gujarat Indians in Houston, TX

^a^ African Americans and European Americans in present study

^b^ HapMap data according to NCBI Entrez database

^c^ Umamaheswaran et al. (9)

^d^ Lee et al. (10)

^e^ 1000 Genomes ASW: Population of African ancestry from southwest USA

Minor allele frequency (in bold)

### Comparison of the D’ linkage disequilibrium (LD) patterns of the *CYP19A1* gene between Arkansas-AA and Arkansas-EA populations

**Fig 1:**
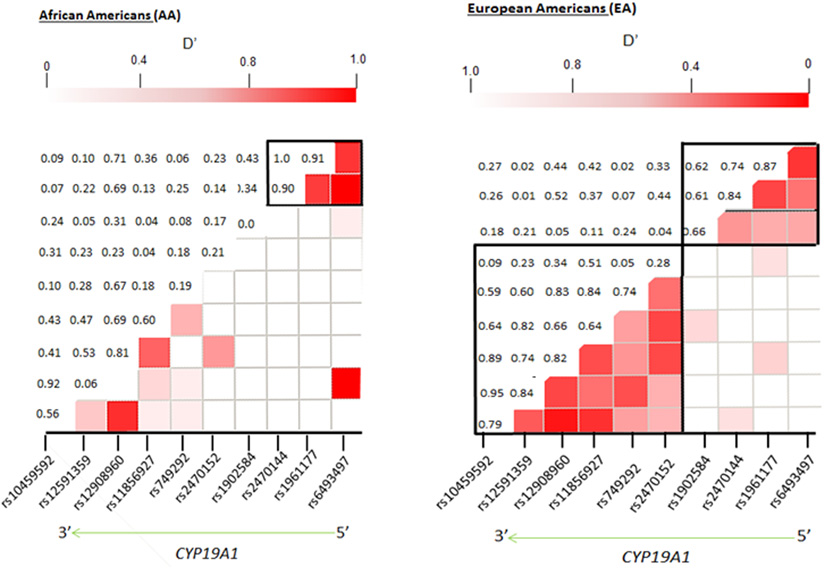
Haplotype block structures for genotyped *CYP19A1* SNPs on chromosome 15q for EA and AA subjects from Arkansas. Shown above are the approximate locations of each of the ten *CYP19A1* SNPs (identified by their dbSNP rs number) among EA and AA populations. The values within the figure refer to the D’ values for each pairwise comparison. The color gradient from red to white indicates higher to lower LD with the darker color indicating higher LD between the SNP pairs.

We made separate D’ LD plots of the *CYP19A1* gene for the EA and AA populations in order to examine the similarities and differences in the LD pattern of *CYP19A1* SNPs between these two populations. Pairwise LD analysis revealed differences in sizes and patterns of LD block between AA and EA populations at the *CYP19A1* gene (Fig. 1). The LD values indicated that there were differences between the two ethnic groups. A relatively strong LD pattern (defined as having a pairwise D’ > 0.8) was observed between rs10459592, rs12591359, and rs1290896; rs6493497, rs1961177 and rs2470144 among EA populations while only rs6493497, rs1961177 and rs2470144 had a strong LD pattern among AA. In EA, six *CYP19A1* SNPs (defined by rs2470152, rs749292, rs11856927, rs12908960, rs12591359, and rs10459592) were also in linkage and resulted in a 58 kb haplotype block. A smaller block of 19 kb (defined by rs6493497, rs1961177, rs2470144, and rs1902584) was also observed in EA while only one much smaller LD block, 9 kb, was observed in AA. The boundary of the two blocks in EA population was between rs1902584 and rs2470152. Therefore, the LD block extended over 70 kb, with 16kb inter-block distance between the two blocks.

### Genetic diversity of *CYP19A1* haplotypes between AA and EA populations

**Fig 2:**
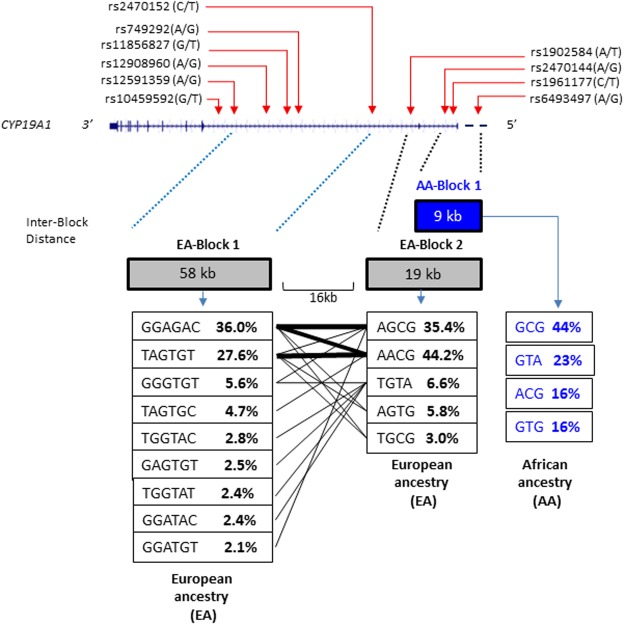
Genomic organization of *CYP19A1* showing the 10 SNPs used in the haplotype analysis. Presented is the *CYP19A1* gene and locations of the 10 *CYP19A1* SNPs on chromosome 15 coordinates 51, 536,349–51,338,598 estimated using UCSC Genome Browser February 2009 (GRCh37/hg19). SNPs are indicated by the rs number highlighted in red. Illustrated below the *CYP19A1* gene are the LD blocks and common haplotypes (≥ 2%) estimated using all SNPs across AA and EA groups separately. The red dotted lines denote the SNPs defined within the corresponding LD block. The lines between blocks link haplotypes that are transmitted with ≥ 2% frequency across blocks. LD blocks constructed in Fig. 1 match haplotype blocks generated in Fig. 2.

Based on the LD patterns, we were able to construct haplotype blocks that are shown in Fig. 2. Towards the 5’ end of *CYP19A1*, we observed the 58 kb EA-specific haplotype block 1 comprising of haplotypes defined by nine different allele arrangements. Towards the 3’ of the gene we observed a larger EA-specific haplotype block 2 defined by four SNPs (with five different allele arrangements) and the only smaller AA-specific haplotype block 1 defined by three SNPs (with four different allele arrangements). The most common haplotypes in EA were AGCG (35%), AACG (44%), GGAGAC (36%), and TAGTGT (27%) while GCG (44%), GTA (23%), ACG (16%) and GTG (16%) were the most common haplotypes in the AA population (Fig. 2).

## Discussion

To our knowledge, this is the first study to report the allele and genotype frequencies, LD pattern, Hardy-Weinberg equilibrium and haplotype structures of *CYP19A1* intron SNPs in populations of African and European ancestry from Arkansas. Arkansas is a primarily rural state that has a high incidence of breast cancer and other health related disparities, particularly among populations of African ancestry. Patterns of genetic variation in the population of Arkansas has been influenced by a regional-specific demographic history (e.g., changes in population size, short- and long- range migration events, admixture and environment) as well as locus-specific forces such as natural selection, recombination, and mutation [Unpublished data] [23]. Thus, we hypothesized that the allele frequency distributions in *CYP19A1*, because of its association with breast cancer and role in estrogen biosynthesis, may be different between populations of African and European ancestry from Arkansas. Therefore, we conducted the present study. For this study, we focused on *CYP19A1* SNPs that were predicted to localize within regulatory binding regions, and/or predicted to associate with regulatory proteins involved in pre-mRNA processing, mRNA metabolism and transport, and previously associated with cancer risk and patient outcomes. Furthermore, the ten *CYP19A1* SNPs genotyped have been reported to influence hormone estradiol levels in postmenopausal women [13,24], predict clinical outcome in metastatic breast cancer patients treated with letrozole [25], and significantly increase risk of colon [26] and endometrial cancer development [13,24]. Due to the clinical impact of CYP19A1 in disease risk, this study was initiated to determine whether stratification by ethnicity would reveal regional-specific significant differences in allele frequencies of *CYP19A1* SNPs between populations of African and European ancestry in Arkansas.

The allele frequencies of six of the ten *CYP19A1* SNPs, rs10459592, rs12908960, rs1902584, rs2470144, rs1961177, and rs6493497, were significantly different between populations of European and African ancestry from Arkansas and when compared to international HapMap populations. Similar findings have also been reported in populations of South Indian (SI) and Korean (KOR) origin, respectively [9,10]. Studies by Umamaheswaran *et al*., [9] and Lee *et al*. [10], demonstrated that the minor allele frequencies for several *CYP19A1* intronic SNPs were significantly different in SI and KOR populations, respectively, compared with HapMap populations of similar ethnicity. Using Taqman SNP genotyping assays on 163 healthy subjects of South Indian origin, Umamaheswaran *et al* observed significant differences in the minor allele frequencies for rs10459592, rs749292, and rs6493497 when compared to HapMap populations [7] and 50 unrelated, healthy Koreans in the study by Lee et al. [10]. These genetic differences in *CYP19A1* between ethnic groups stress the importance for considering ancestral differences when determining causal SNPs for disease association studies.

For instance, Haiman *et al* demonstrated that the rs749292 *CYP19A1* SNP was a predictor of circulating estrogen levels in white women of primarily European descent [13]. In the Breast and Prostate Cancer Cohort Consortium (BPC3), a large collaborative prospective study of over 8,000 prostate cancer cases and 9,000 age and ethnicity-matched controls consisting of EA, Latinos, Japanese Americans, and Native Hawaiians, several haplotype tagging SNPs, including rs749292, were found to be in LD and were significantly associated with a 5% to 10% difference in estradiol concentrations in men [16]. Another population-based case-control study of colon cancer patients of European descent showed that individuals homozygous for the “A” minor allele of rs12591359 were associated with an increased risk of colon cancer (OR 1.44 95% CI 1.16–1.80) and rs2470144 was associated with reduced risk of rectal cancer [26]. In our study, we did not observe a significant difference in the minor allele frequency for rs749292 and rs12591359 between our AA and EA populations. This implies that circulating estrogen levels may not be significantly different between ethnic groups with similar minor allele frequencies; however, it is highly unlikely that only one SNP would give rise to a given phenotype. On the other hand, the minor allele frequency for rs2470144 was significantly different between AA and EA and between YRI and JPT international HapMap populations. We also showed that frequencies were similar between CEU and EA groups, but interestingly not between AA and YRI. Recently, the rs10459592 SNP was significantly associated with higher clinical benefit rate from letrozole, an aromatase inhibitor, in 109 Korean hormone receptor positive metastatic breast cancer patients [27], which further supports the importance of investigating tagged SNPs in ethnically diverse populations.

Stratification of AA and EA populations by ethnicity and region also revealed significant differences in haplotype frequencies and LD patterns that were unique to EA and also those common to both populations. Two large LD blocks of 58Kb and 19Kb were observed among EA while a smaller 9Kb LD block was observed in AA. The haplotype blocks clustered in smaller blocks among AA population compared to the EA population, which showed evidence of haplotypes clustering in larger blocks. This feature is most likely attributed to populations of African ancestry that have higher effective population size and genetic diversity. Furthermore, similar LD patterns in the human genome across populations have also been reported previously [27,28]. Therefore, we expected to observe larger LD blocks for EA compared to AA from Arkansas. In our analyses, the AA-specific block 1 harboring GCC (AA-block 1, Fig. 2) is unique and found in both a very large and smaller clades arising from unique common ancestry. It is clear that understanding the allele profiles upstream of AA-block 1 can help map and understand the influence of adjacent SNPs along the haplotypes with regards to severity of certain phenotypes. Similarly EA-specific blocks 1 and 2 (Fig. 2) harbor haplotypes that may provide EA-specific adjacent genetic information for mapping purposes and behavior of phenotypes.

In summary, our results provide evidence of ethnic differences in the frequency of *CYP19A1* SNPs between populations of African and European ancestry from Arkansas. *CYP19A1* is critical for estrogen biosynthesis, thus, identifying genetic variants in *CYP19A1* is necessary for assessing cancer risk and predicting response to aromatase inhibitor drugs across ethnically diverse and disparate populations. Furthermore, because *CYP19A1* contains thousands of intronic SNPs some of which may lie in regulatory regions that may independently alter the function of the enzyme, analysis of low-frequency SNPs and identifying rare haplotypes among ethnic groups that have higher disease risk is critical for understanding the influence of SNPs which define an individual’s genetic background within or adjacent to functional domains that may influence drug response and disease risk.
